# Hypothesis on the pathophysiology of syringomyelia based on analysis of phase-contrast magnetic resonance imaging of Chiari-I malformation patients

**DOI:** 10.12688/f1000research.72823.1

**Published:** 2021-10-01

**Authors:** Han Soo Chang

**Affiliations:** 1 Department of Neurosurgery, Tokai University, 143 Shimokasuya, Isehara, Kanagawa, 259-1143, Japan

**Keywords:** syringomyelia, Chiari malformation, pathophysiology, hypothesis, magnetic resonance imaging, phase-contrast

## Abstract

**Background:** Despite a number of hypotheses, our understanding of the pathophysiology of syringomyelia is still limited. The current prevailing hypothesis assumes that the piston-like movement of the cerebellar tonsils drives the cerebrospinal fluid (CSF) into the syrinx through the spinal perivascular space. However, it still needs to be verified by further experimental data. A major unexplained problem is how CSF enters and remains in the syrinx that has a higher pressure than the subarachnoid space.

**Methods:** I analyzed phase-contrast MRI scans of 18 patients with Chiari-I malformation with syringomyelia undergoing foramen magnum decompression and 21 healthy volunteers. I analyzed the velocity waveforms of the CSF and the brain in various locations. The obtained velocity waveforms were post-processed using a technique called *synchronization in situ*. I compared between the preoperative data and the control data (case-control study), as well as between the preoperative and postoperative data (cohort study).

**Results:** The syrinx shrank in 17 (94%) patients with good clinical improvement. In Chiari-I patients, the velocity of the tonsil was significantly larger than controls, but was significantly smaller than that of the CSF in the subarachnoid space, suggesting passive rather than active movement. The abnormal tonsillar movement disappeared after surgery, but the velocity waveform of the spinal subarachnoid CSF did not change. These results, contradicting the above mentioned hypothesis, required an alternative explanation. I thus hypothesized that there is a CSF channel between the fourth ventricle and the syrinx. This channel assumes one-way valve function when mildly compressed by the cyclical movement of the cerebellar tonsil. The decompression of the tonsils switches off the one-way valve, collapsing the syrinx.

**Conclusions: **My hypothesis reasonably explained my data that clearly contradicted the existing hypothesis, and successfully addressed the above-mentioned theoretical problem. It will serve as a working hypothesis for further study of syringomyelia pathophysiology.

## Introduction

Syringomyelia is a disease in which a fluid-filled cavity, a syrinx, is formed inside the spinal cord causing neurological symptoms. The mysterious pathophysiology of syringomyelia has fascinated many researchers for decades. Various authors have proposed a number of hypotheses to explain the mechanism of syrinx formation. ^1- 13^ However, these hypotheses contradict one another, and none seems to fully explain the pathophysiology of syringomyelia.

The current prevailing hypothesis of Oldfield *et al*., proposes that piston-like movements of cerebellar tonsils produce pressure waves in the cerebrospinal fluid (CSF), which drive CSF into the syrinx through the perivascular space. ^14^ Despite being attractive, it is still a hypothesis and needs to be verified by new experimental data.

Above all, a fundamental question seems to remain unexplained. It is evident that the pressure inside the syrinx is higher than that of the CSF outside, which is required by the laws of physics ^15^ and has been shown by direct measurements. ^7, 16, 17^ No hypothesis so far, however, has explained how CSF enters and remains in the syrinx, which has higher pressure than the outside subarachnoid space.

In this article, I present my data obtained by detailed analysis of phase-contrast magnetic resonance imaging (MRI) of patients with syringomyelia associated with Chiari-I malformation. Based on these data, I propose a new hypothesis on the pathophysiology of syringomyelia, which attempts to address the above-mentioned problem.

## Methods

### Material

In this study, I dealt solely with patients with Chiari-I malformations who had associated syringomyelia in the cervical cord. The following patients were excluded: those with syringomyelia not related to Chiari-I malformation, those with syringomyelia with basal arachnoiditis, and those with Chiari-I malformation without syringomyelia.

This study, which was approved by the Institutional Review Board of Tokai University Hospital. No. 18-609, was a retrospective study on prospectively acquired data. Since January 2011, I have routinely incorporated phase-contrast studies into the cervical spine MRI of patients with Chiari-I malformation. The MRI studies were performed before surgery and at each postoperative follow-up visit, namely, at six months postoperatively, one year postoperatively, and then at intervals of one year postoperatively. These MRI studies used the same acquisition techniques described below.

From January 2011 to April 2019, a total of eighteen compatible patients underwent foramen magnum decompression at our institution. These patients were enrolled in my study consecutively during the study period. Because one patient’s preoperative data was missing, we analyzed 17 preoperative MRI and 18 postoperative MRI. At the same time, I recruited 21 healthy volunteers from our hospital staff so that the mean age would be comparable with that of the patients. They underwent the same MRI studies.

### Surgery

All patients underwent foramen magnum decompression performed by the first author using the same surgical techniques, which consisted of a small suboccipital craniotomy of 2 × 2 cm, C1 laminectomy, and a Y-shaped dural incision followed by fascia patching. I did not perform intradural exploration, which was not necessary in the studied population with no basal arachnoiditis. Then, I replaced the free suboccipital bone flap over the decompressed dura with titanium miniplates, adjusting its angle so that it would not compress the dura. Then, I sutured the fascia patch on the edge of the bone flap. This manoeuvre maintained decompression by preventing postoperative scar tissue invasion.

### MRI method

All magnetic resonance images were obtained using a 1.5 Tesla scanner (Achieva, Philips Medical System, Best, The Netherlands). At each MRI session, phase-contrast images were obtained in the midline sagittal section with the movement in the cranio-caudal direction encoded into intensity with a velocity encoding (VENC) of 10 cm/sec. Data acquisition was triggered by the QRS wave of the patient’s electrocardiogram with the cardiac cycle divided into 25 or 35 segments. The detailed imaging parameters were as follows: TR 16 msec, TE 7.2 msec, flip angle 15 degrees, field of view 256 × 256, matrix 352 × 256, and slice thickness 5 mm.

**Figure 1.**
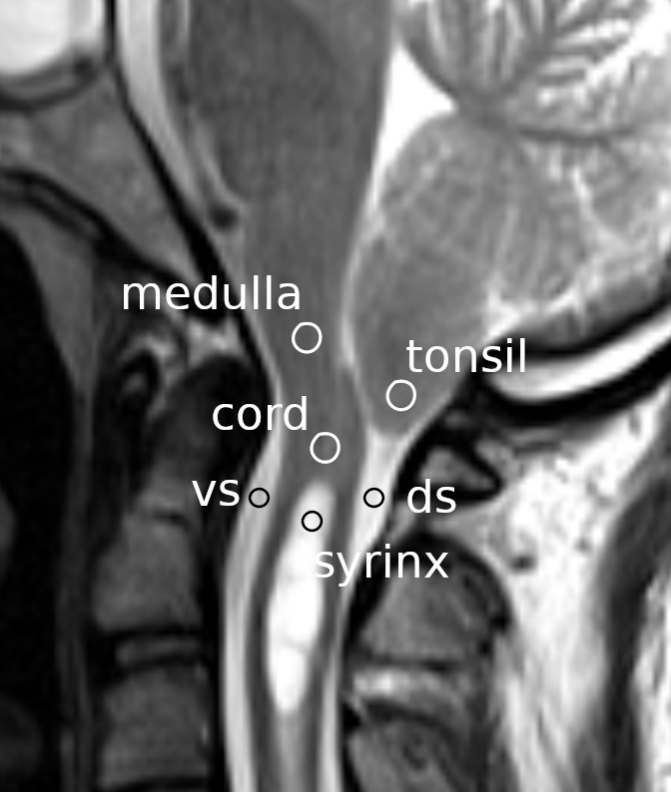
Regions of interest measured. Circles indicate the regions of interest on a mid-sagittal section of a magnetic resonance image at the craniovertebral junction. vs: ventral subarachnoid space; ds: dorsal subarachnoid space.

The phase-contrast images of each subject were displayed on a computer monitor using an image-processing application ( ImageJ version 1.52a, National Institutes of Health, Bethesda, Maryland, United States (RRID:SCR_003070)). Circular shaped ranges of interest (ROIs) were set at the following locations ( Figure 1): the cerebellar tonsil, the spinal cord segment between the fourth ventricle and the syrinx, the ventral subarachnoid space at the level of the base of the odontoid process, the dorsal subarachnoid space immediately below the tonsil, the rostral portion of the syrinx cavity, and the medulla at the level slightly above the foramen magnum. In the controls, the same ROI settings were used except for the ROI at the syrinx, which was set instead at the cord segment at the level of the C3 vertebral body. The average flow speed of the pixels inside each ROI was measured at each time point of the cardiac cycle. Thus, six waveforms corresponding to the six ROIs were obtained for each MRI session and were stored in a computer file.

***Synchronization in situ***

The obtained data started at the QRS wave of the electrocardiogram of each patient. However, the latency from the QRS wave to the initial rise of brain and CSF movements significantly varied among patients. This created difficulty when I tried to analyze the data in more detail. I needed to post-process the data so that the initial rise of the brain and CSF movements would be better synchronized. For this purpose, I developed a post-processing technique that I called *synchronization in situ.*

First, I defined the CSF trigger point as the time point where the ventral subarachnoid CSF started to move caudally i.e. where the CSF velocity most rapidly changed in the caudal direction. The CSF trigger point could be identified for each MRI session using the ventral subarachnoid CSF waveform contained in the data file. Then, I standardized/synchronized the six waveforms of each MRI session in the following manner. First, to standardize the different number of time bins among the MRI sessions, I increased the number of time bins per cardiac cycle to 50 using linear interpolation. I then shifted and synchronized the six waveforms using ring buffers so that the CSF trigger point would become the middle point of the waveform. The post-processed data is available in a data repository. ^18, 19^ The software used for the data analysis is available in a software repository. ^19, 20^

### Data analysis

I compared the following three groups: (1) Preoperative studies of Chiari-I patients, (2) Studies of normal volunteers, and (3) Postoperative studies of Chiari-I patients at the last clinical visit. For each group, the mean velocities at the six locations were calculated at each time point in the cardiac cycle. The obtained waveforms were plotted for each group.

I statistically compared the peak caudal velocity at each ROI. Comparisons were made between the controls and the preoperative Chiari-I patients and between the preoperative and postoperative Chiari-I patients. For the ROI of the spinal cord, I also compared the peak rostral velocity in addition to the caudal velocity because, as I describe below, paradoxical rostral movement of the spinal cord was observed in Chiari-I patients.

For the statistical analyses, I used the unpaired t test for the comparison between the controls and the preoperative Chiari-I patients and the paired t test for the comparison between the pre- and postoperative Chiari-I patients. For the comparisons of tonsillar velocity and CSF velocities in the dorsal and ventral subarachnoid space, I used analysis of variance with Tukey’s post hoc multiple comparison test. P values smaller than 0.05 were considered statistically significant.

The following computer packages were used for the statistical analyses: R version 4.0.4 (R Project for Statistical Computing, RRID:SCR_001905) and RStudio version 1.2.1 (RStudio, RRID:SCR_000432).

## Results

### Clinical results

The mean and standard deviation (SD) of the age of the patients was 40.2 years (SD 16.0). There were fourteen female and four male patients. The mean (SD) age of the volunteers was 33.7 years (SD 9.8). There were seven female and fourteen male volunteers.

**Figure 2.**
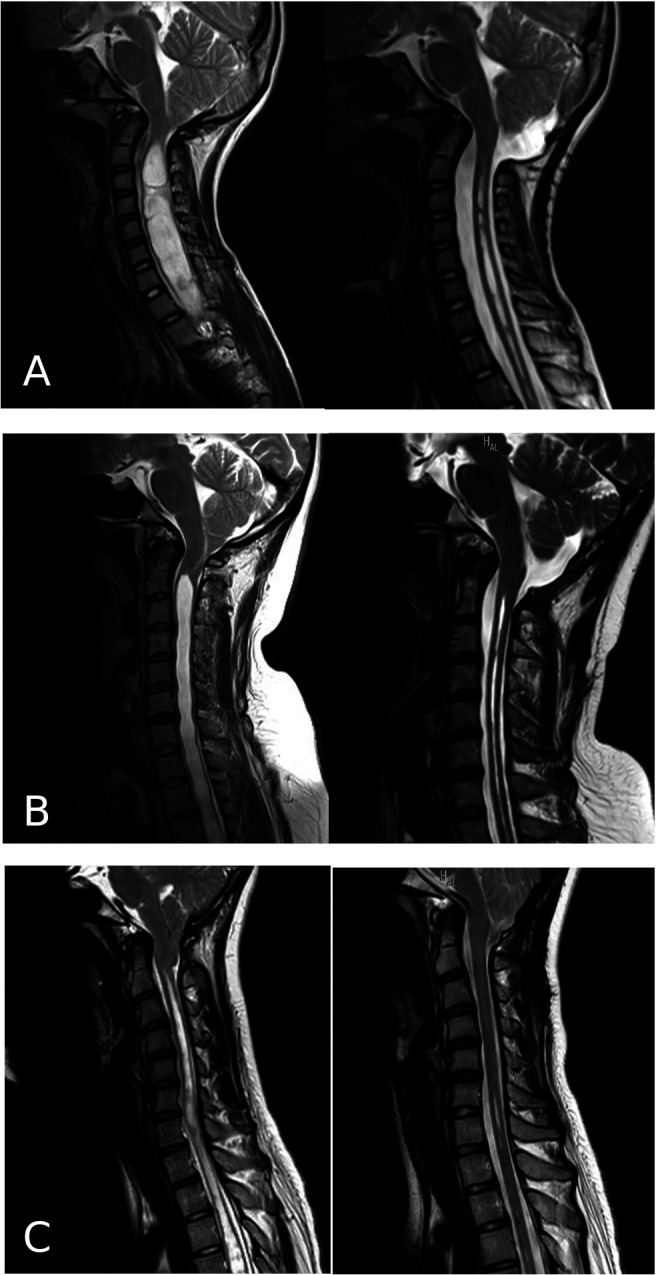
Pre- and postoperative cervical-spine MRI of three representative cases (A, B, and C). The left column shows the preoperative images, and the right column shows the postoperative images.

There were no surgery-related complications. The mean period from surgery to the final postoperative MRI was 633 days (SD 555, range 70–1700). The syrinx shrank in seventeen out of the eighteen patients (94%) with improvement of preoperative symptoms ( Figure 2). In one patient, upper extremity pain persisted after surgery despite shrinkage of the syrinx.

### Velocity waveforms of preoperative Chiari-I patients

**Figure 3.**
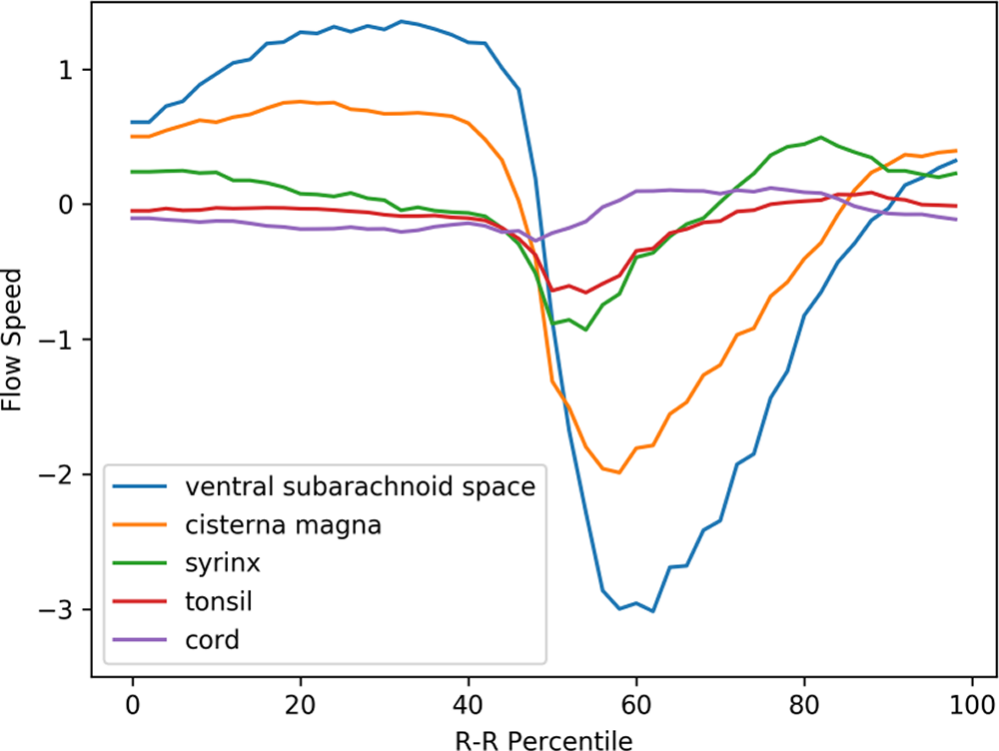
Mean velocity waveforms in preoperative Chiari-I patients. The abscissa shows one cardiac cycle divided into 100% percentile. The ordinate shows the flow speed (cm/sec). The waveforms are synchronized so that the maximal caudal acceleration of the ventral CSF is placed at the 50th percentile

Figure 3 shows the mean velocity waveforms of the five ROIs (the medulla, which made almost no movement, was excluded) in preoperative Chiari-I patients. As shown in the figure, the cerebellar tonsil (red line) made a rapid caudal movement in synchrony with the rapid caudal flow of the cerebrospinal fluid in the ventral and dorsal subarachnoid space (blue and yellow line). The timing of this tonsillar movement was in synchrony with that of CSF movement ( Figure 3).

The peak caudal velocity of the tonsils was significantly smaller than that of the CSF in the subarachnoid space. The mean (SD) of the peak caudal velocity was 0.76 (0.47), 2.3 (1.7), and 3.5 (2.0) for the tonsils, dorsal subarachnoid space, and ventral subarachnoid space, respectively. These differences were statistically significant (F = 13.9, p < 0.001), and Tukey’s post hoc test showed that the difference of each comparison pair was statistically significant with p values of 0.02, <0.001, and 0.05 for the tonsil vs. dorsal subarachnoid space, the tonsil vs. ventral subarachnoid space, and dorsal subarachnoid space vs. ventral subarachnoid space, respectively.

The syrinx fluid (green line in Figure 3) also made a rapid caudal movement in synchrony with the CSF and the tonsil. This fluid movement appeared early in the time course and was almost simultaneous with the caudal flow of the CSF ( Figure 3). There was no noticeable delay or phase shift between the commencement of caudal syrinx fluid movement and that of the subarachnoid CSF movement ( Figure 3). On the other hand, the reverse flow in the cranial direction began much earlier inside the syrinx than in the subarachnoid space ( Figure 3).

**Figure 4.**
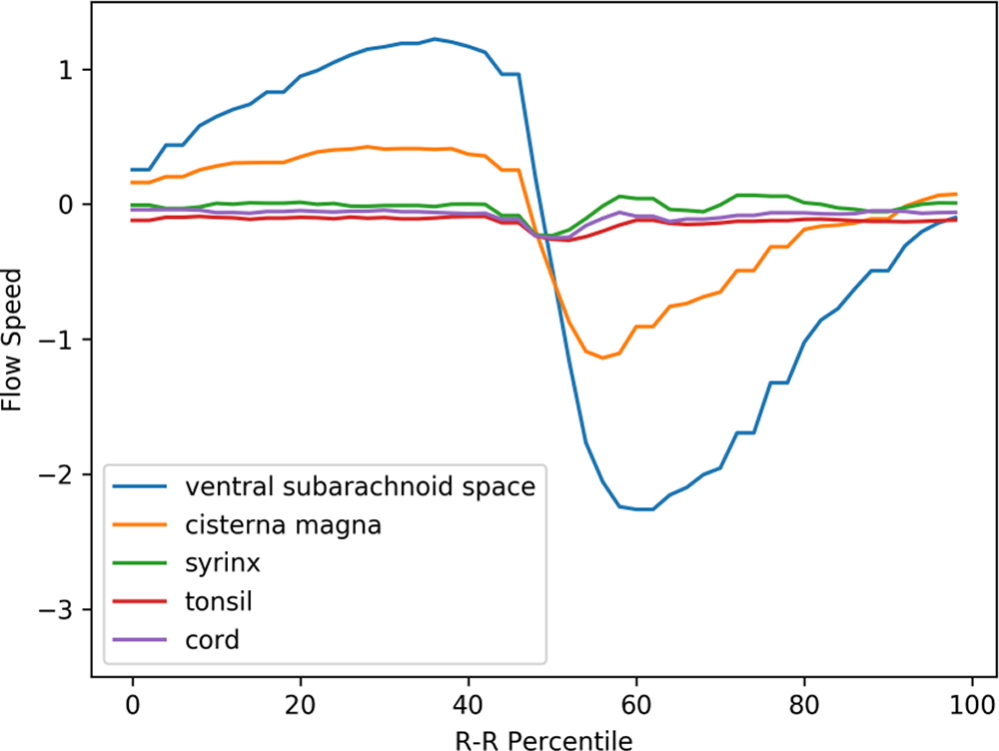
Mean velocity waveforms in controls. The same abscissa and ordinate as in Figure 3. (The legend *syrinx* denotes the spinal cord at the C5 level.)

A puzzling finding shown in Figure 3 was the rostral movement of the upper cervical cord (purple line) when all the other parts were moving caudally in patients. This paradoxical movement of the cord was barely seen in the controls ( Figure 4). The difference was statistically significant ( Table 1).

**Table 1.** Peak caudal velocity at the ROI in the three groups.

	Control	P (control vs. preop)	Preop	P (preop vs. postop)	Postop
tonsil	0.31 (0.14)	0.001*	0.76 (0.47)	0.002*	0.32 ( 0.39)
ventral SA	2.5 (0.77)	0.06	3.5 (2.0)	0.82	3.4 (1.3)
dorsal SA	1.3 (0.61)	0.04*	2.3 (1.7)	0.34	1.9 (1.1)
syrinx	0.34 (0.19)	0.001*	1.4 (1.1)	0.24	1.1 (1.0)
cord (rostral)	−0.0087 (0.0085)	0.002*	−0.31 (0.25)	0.49	−0.46 (0.34)
cord (caudal)	0.34 (0.13)	0.06	0.51 (0.32)	0.31	0.39 (0.23)
medulla	0.31 (0.11)	0.40	0.34 (0.25)	0.13	0.31 (0.20)

### Comparison between preoperative Chiari-I patients and controls

The prominent movement of the cerebellar tonsils seen in preoperative Chiari-I patients (red line in Figure 3) was absent in controls (red line in Figure 4). Paradoxical rostral movement of the upper cervical cord (purple line in Figure 3) observed in patients was also barely seen in the controls (purple line in Figure 4). These differences were statistically significant ( Table 1). The CSF velocities in the subarachnoid space were also significantly larger in preoperative Chiari-I patients than in controls ( Table 1).

### Comparison between pre- and postoperative Chiari-I patients

**Figure 5.**
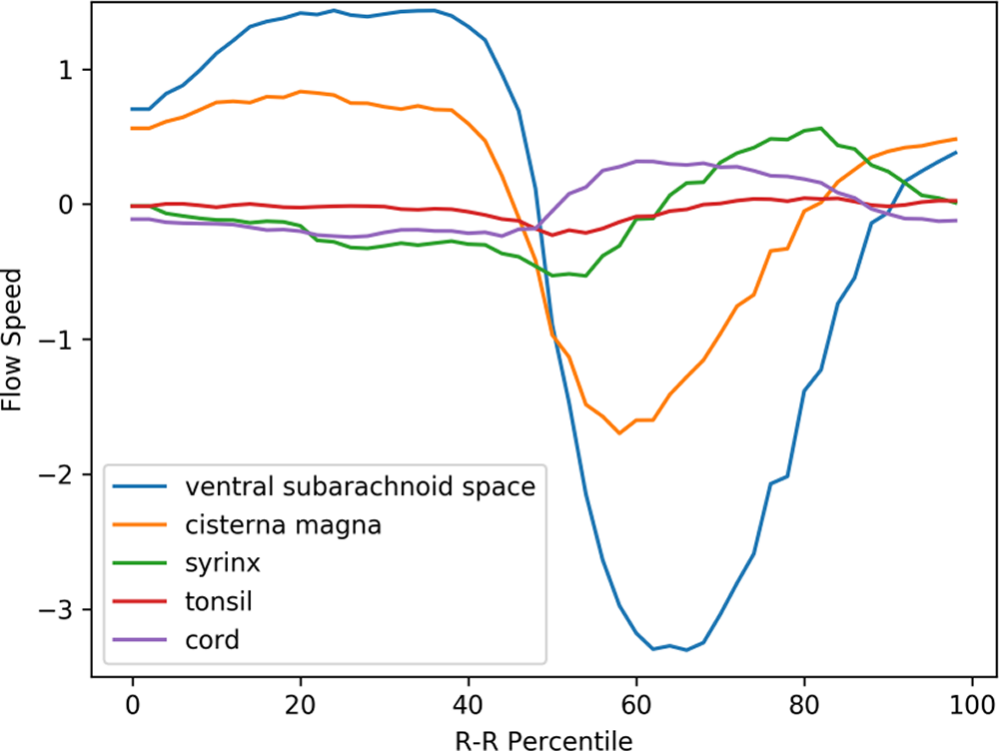
Mean velocity waveforms in postoperative Chiari-I patients. The same abscissa and ordinate as in Figures 3 and 4.

**Figure 6.**
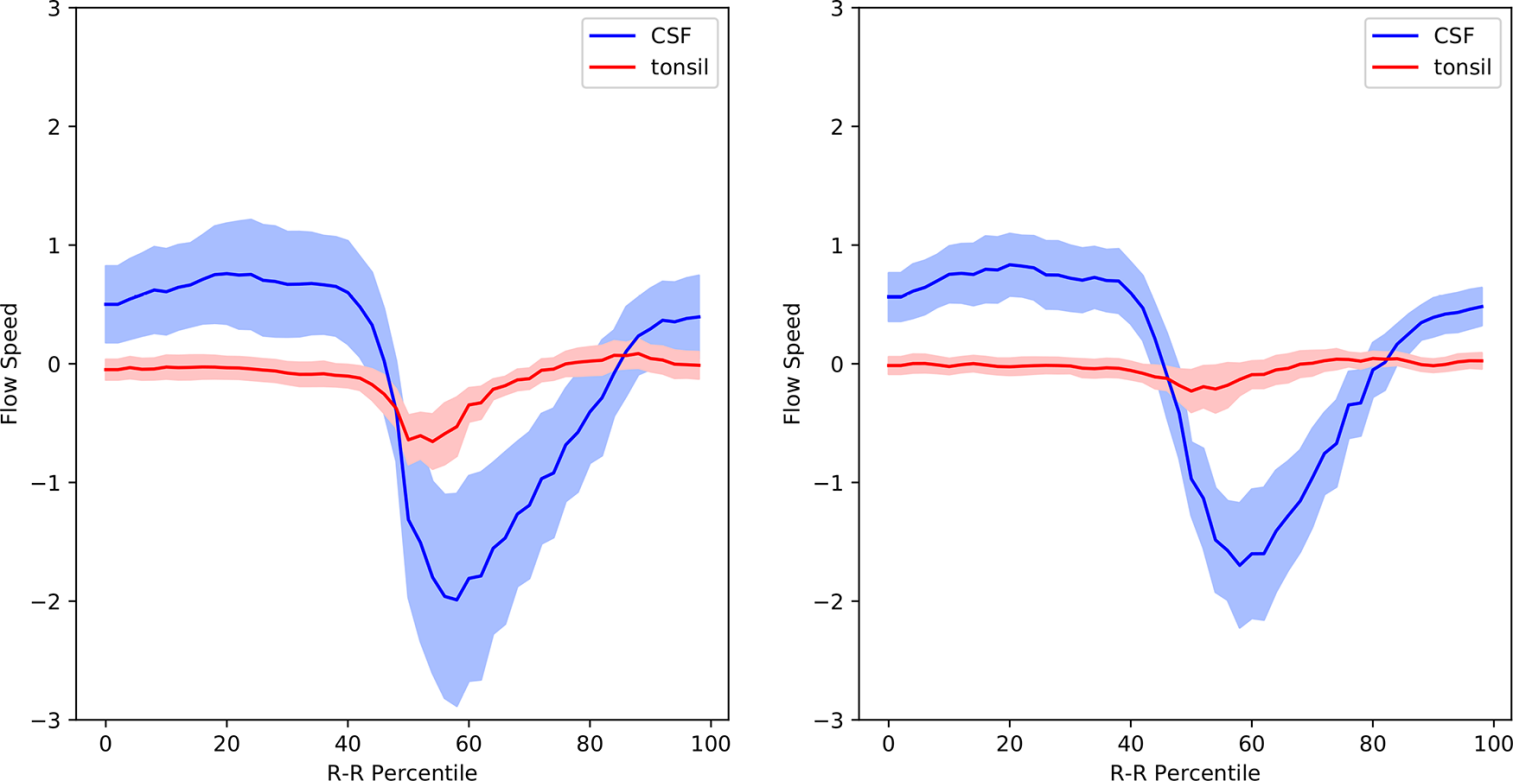
Velocity waveforms of the tonsil and the dorsal CSF in pre- and postoperative Chiari-I patients. The mean value is shown together with the 95% confidence intervals.

Figure 5 shows the postoperative mean velocity waveforms. The most notable postoperative change was disappearance of tonsillar movement. On the other hand, the velocity profiles of the other areas analyzed did not significantly change from the preoperative profiles ( Figures 3, 5, Table 1). Figure 6 shows the velocity waveforms of the tonsil and the dorsal CSF extracted from Figures 3 and 5 together with their 95% confidence intervals. It can be clearly seen that the tonsillar velocity was significantly smaller than the CSF velocity, and despite the postoperative disappearance of tonsillar movement, the CSF velocity did not change significantly ( Figure 5, Table 1).

## Discussion

### Summary of my findings

In preoperative Chiari-I patients, the velocities of the cerebellar tonsil and spinal subarachnoid CSF were significantly elevated compared to those of controls ( Figures 3, 4, Table 1). This finding agrees well with other studies in the literature. ^10, 21- 25^ I could, however, clearly show that the velocity of the tonsil was much smaller (approximately one-third) than that of the subarachnoid CSF ( Figure 6, Table 1).

The abnormal tonsillar movement disappeared after surgery ( Figures 5, 6). This also agrees with previous studies. ^7, 26, 27^ However, the postoperative subarachnoid CSF velocities remained elevated in my data ( Figure 5, Table 1). Regarding this point, the literature showed conflicting results; the postoperative CSF velocity either decreased, increased, or remained unchanged. ^7, 26, 27^ This variability may have been caused by some of the differences in surgical techniques such as the size of the craniectomy. The important point is that the syrinx shrank in 94% of my patients in whom CSF velocity postoperatively remained unchanged.

### Data interpretation

According to the hypothesis of Oldfield *et al*., ^7, 14^ the piston-like movement of the tonsil generates pressure waves in the spinal subarachnoid CSF, which drives the CSF into the syrinx through the perivascular space of the cord. However, these results cannot be interpreted in accordance with this hypothesis. First, my data clearly showed that the velocity of the tonsil was much smaller than that of the CSF ( Figure 6). It is difficult to imagine that a slower moving object can become the source of a faster moving object. Second, the velocity of the subarachnoid CSF did not decrease after surgery despite the postoperative disappearance of tonsillar movement ( Figure 6). If the increased CSF pressure is the cause of a syrinx, it is difficult to explain the shrinkage of syrinxes in my patients while the CSF velocities were unchanged postoperatively.

Therefore, these data can perhaps be interpreted more naturally as follows. The herniated tonsils in Chiari-I patients reduced the cross-sectional area of the subarachnoid space at the craniovertebral junction. Consequently, the velocity of the CSF at the craniovertebral junction was elevated because of the so-called Venturi effect. ^15^ The piston-like movement of the cerebellar tonsil is rather the result, not the cause, of this increased CSF velocity at the craniovertebral junction.

In that case, how does the abnormal tonsillar movement in Chiari-I patients relate to syrinx formation? Because it was the only parameter that changed when the syrinxes shrank, the abnormal tonsillar movement may well be closely associated with the pathophysiology of syringomyelia. It did not seem, however, that the two phenomena (tonsillar movements and syrinx formation) were mediated by CSF pressure waves in the subarachnoid space as postulated by Oldfield *et al*.

### Premises of the theory

To better interpret these data, I made the following three premises, some of which may be rather controversial.

1. The origin of the syrinx fluid is the CSF, and there is a channel connecting the syrinx cavity and the subarachnoid space.
2. The central canal cannot be ruled out as a candidate for this channel.
3. There must be some kind of one-way-valve mechanism to sustain the expanded state of the syrinx.

A significant amount of evidence supports the first assumption. The composition of the syrinx fluid is the same as that of the CSF. ^28^ Intrathecally administered contrast or tracer materials readily enter the syrinx cavity. ^29, 30, 31^ Recently, Heiss *et al*., quantitatively analyzed the accumulation of intrathecally administered contrast material into the non tumor-related syrinx. ^30^

The second assumption is controversial and needs to be discussed in detail in the next section.

### The central canal myth

Gardner and Angel ^1^ and Williams ^32^ originally assumed that CSF enters the syrinx through a patent central canal. However, this idea recently lost favor for several reasons.

1. MRI scans do not show the communication between the fourth ventricle and the syrinx in most cases.
2. Autopsy studies of syrinx patients presumably showed little relationship between the syrinx and the central canal.
3. Autopsy studies of patients showed that the central canal progressively becomes obliterated as age increases.

The first point may be an invalid argument. The diameter of the central canal is in the order of 100 μm. ^33^ The current resolution of MRI scans cannot clearly show a channel of this size. ^34^ Therefore, the fact that MRI scans do not portray the communication between the fourth ventricle and the syrinx may prove nothing about the presence or absence of such a channel.

As to the second point, Milhorat *et al*., ^35^ published the largest autopsy series of 105 syrinx patients. The authors classified the cases into 47 communicating and 23 noncommunicating syrinxes based on the MRI findings. The other 35 cases were syrinxes of various etiologies. In these 23 noncommunicating syrinxes, 70% rostrally continued to a stenotic central canal and 30% continued to a patent central canal. According to the author’s description, the stenotic central canals did not seem to be obliterated. Simply put, 100% of the noncommunicating syrinxes rostrally continued to a patent central canal. Therefore, these data support, rather than exclude, the possible role of the central canal in the pathogenesis of syringomyelia.

Regarding the third point, the central canal was conventionally deemed to be occluded in human adults. ^34^ However, recent studies have shown that this occlusion is an age-related gradual process. ^33, 36^ Newman *et al*., ^36^ found in their study of 60 autopsy cases that the central canal was patent up to the fourth decade of life, while Yasui *et al*., ^33^ found that occlusion of the central canal occurred in somewhat earlier ages. Milhorat *et al*. reported in their study of 232 autopsy cases that occlusion of the central canal was seen only in four individuals. ^37^ Storer *et al*., ^34^ considered that ”the study of the morphology of the central canal is difficult using only histological sections” because of its three-dimensional characteristic and proposed a computerized 3-D method of evaluation. Considering that Chiari-I malformation occurs in the pediatric population and in relatively early adulthood, ^3, 38^ the possibility that a patent central canal plays an important role in the pathogenesis of syringomyelia cannot be ruled out.

### One-way valve mechanism

The third assumption was that there must be a one-way valve mechanism to explain the generation and maintenance of syringomyelia. This idea has been expressed by previous authors. ^6, 39^ However, the concept of the one-way valve mechanism has not been duly incorporated in the previous theories of syringomyelia. This concept, however, may turn out to be essential if I consider the following points. First, basic physical laws indicate that the syrinx in its distended state has higher pressure inside than outside. ^15^ It can be intuitively understood that the inside pressure must resist the elastic force of the syrinx wall in addition to the outside pressure. A few experiments with direct measurement of the syrinx pressure have proven that interior pressure was higher than the outside pressure. ^17, 39^

Therefore, any theory of syringomyelia assuming a CSF channel between the syrinx and the subarachnoid space must explain the mechanism by which the syrinx maintains its expanded state against the pressure gradient. Without this mechanism, CSF will flow out from the higher-pressure syrinx cavity to the lower-pressure subarachnoid space until the syrinx is collapsed and the pressure gradient is equilibrated. Assuming the existence of a one-way valve is a reasonable idea to solve this problem.

### Hypothesis

My hypothesis on the pathophysiology of syringomyelia can be summarized as follows:

1. There is a communicating channel between the fourth ventricle and the syrinx, most likely a patent central canal.
2. This channel assumes a one-way-valve function when mildly compressed by herniated tonsils.
3. Repetitive pressure waves pump CSF through this valve, thereby creating a syrinx distally.
4. The foramen magnum decompression switches off this one-way valve by removing the local compression, leading to the collapse of the syrinx.

The idea of the central canal working as a one-way valve was proposed by du Boulay *et al*., in 1974. ^6^ This long-forgotten idea needs to be reviewed in the context of the premises described above. The herniated tonsils in Chiari patients move like a piston in accordance with the cyclic CSF movements. I may analogize this piston-like movement to that of a ball in a ball valve. In other words, the CSF undergoes more resistance in the caudal direction than in the rostral direction. The data showed that the velocity of the tonsils and the CSF near the craniovertebral junction has higher peak velocity in the caudal direction than in the rostral direction ( Figure 3). Because higher velocity means higher resistance according to the Venturi effect, the data suggest that there is unidirectional resistance to the CSF flow at the craniovertebral junction; namely, the resistance to the caudal CSF flow is higher than that to the rostral CSF flow. This phenomenon has been demonstrated by Williams *et al*., and he postulated that this unidirectional CSF resistance gives rise to a *sucking* mechanism that generates syrinxes. ^2, 32^ My hypothesis somewhat resembles Williams’ idea, but more detailed explanation is needed. I showed in our previous article ^12^ that, when resistance to CSF flow in the subarachnoid space is increased at some point, the transmural pressure in the central canal is increased in the downstream segment. Therefore, with reciprocating flows across a point with unidirectionally increased resistance, increased intramural pressure will be repetitively generated in the central canal situated downstream to the increased resistance. This will in effect bring forth a one-way valve mechanism in the central canal. I have supporting evidence for this idea based on our simulation model ^11, 12^ and will report it in our future correspondence. My hypothesis can also clearly explain why the limited decompression at the foramen magnum is effective in shrinking the syrinx. By localized decompression at the craniovertebral junction, the compression of the cord is relieved, and the piston-like movement of the tonsils ceases together with the unidirection CSF resistance. This will turn off the one-way valve function of the central canal. The syrinx fluid will then flow out according to the pressure gradient, eventually collapsing the syrinx. My hypothesis solves the problem concerning the interpretation of my phase-contrast data. The only difference in the postoperative phase-contrast data was the disappearance of the tonsillar movement; there was no significant difference in the movement of the CSF in the subarachnoid space ( Figure 6). Therefore, an explanation was needed about how the cessation of tonsillar movement caused the syrinx to shrink without significant changes in subarachnoid CSF movement. My hypothesis explains it in a straightforward way. The disappearance of the piston-like movement of the tonsils cancels the ball-valve mechanism functioning in the central canal. The subarachnoid CSF movement has no role in this causal relationship.

On the other hand, other hypotheses that regard the perivascular space as the CSF channel may face two major problems. First, these hypotheses will have difficulty identifying a one-way valve. There are no structures along the perivascular space that would function as a one-way valve. Nevertheless, if I cannot assume a one-way valve mechanism, the CSF in the syrinx will flow out according to the pressure gradient between the syrinx and the subarachnoid space. Second, even if there is a one-way valve in the perivascular space in the spinal cord, how does this valve cease to function after simple decompression at the craniovertebral junction? This may be a difficult question to answer. Thus, in my opinion, the perivascular space theories contain a serious theoretical problem.

### Paradoxical rostral movement of the spinal cord

I reported here the paradoxical rostral movement of the upper cervical cord in Chiari-I patients. This movement can be observed in the movie of color-coded phase-contrast data of one representative patient. ^19, 40^ This phenomenon has not been previously reported in the literature. I only found similar rostral movement in a figure in the article by Hofman *et al*., ^23^ who studied Chiari-I patients using phase-contrast MRI. The authors, however, did not discuss this phenomenon or perform statistical analysis.

The phenomenon is paradoxical because all the other parts (ventral and dorsal CSF, cerebellar tonsils, and syrinx fluid) are moving caudally, while the cord segment between the fourth ventricle and the syrinx is moving rostrally. For this paradoxical cord segment movement to occur, there must be some force exerted on it. This force must be exerted by some adjacent tissue. The medulla makes almost no movement during this period, and the outside CSF is moving rapidly downward. The only possible source of this force may be the syrinx fluid on the caudal side of this cord segment. If I assume some CSF movement from the fourth ventricle to the syrinx through the central cord or some other channel, this phenomenon may become somewhat more comprehensible. Further studies will be needed to carefully examine this phenomenon.

### Shortfalls and future prospects

The number of patients was relatively small. Although I obtained statistically significant results, the data might well be interpreted with a certain amount of caution. Although my hypothesis avoided the difficulty of existing hypotheses and explained my obtained data reasonably well, it still lacks sufficient direct evidence to claim its veracity. My hypothesis thus remains a hypothesis until further supporting evidence is obtained in the future. It will, however, serve as a working hypothesis for future studies of syringomyelia. The exact mechanism how the one-way valve mechanism appears in the central canal when the outside CSF movement is blocked in one direction is not described in this report. I am currently studying this point using computer simulation. The results of this study will be reported in a future article. Regarding the paradoxical rostral movement of the spinal cord, its velocity was relatively small. This interesting phenomenon needs further careful study. If a similar type of block of the CSF movement as caused by the cerebellar tonsil occurs in other locations in the spinal canal, syrinxes may be generated with the same mechanism. It is thus possible that my hypothesis be extended to explain the pathophysiology of syringomyelia associated with arachnopathy. ^41^

## Conclusions

The analysis of phase-contrast data of Chiari-I patients showed data that contradicted the existing hypothesis on the pathophysiology of syringomyelia. My hypothesis that the central canal assumes a one-way valve function when compressed by the cyclical movement of the cerebellar tonsils could reasonably explain the data and further explain how the CSF enters and remains in the syrinx that has higher pressure than the outside subarachnoid space.

## Data availability

### Underlying data

Dryad: Underlying data ‘Phase-contrast MRI data of 18 Chiari-I malformation patients and 21 controls’. https://doi.org/10.5061/dryad.37pvmcvm0. ^18^

- Data files: encoded_data.json

Data are available under the terms of the Creative Commons Zero “No rights reserved” data waiver (CC0 1.0 Public domain dedication).

### Extended data

Zenodo: Extended data ‘Phase-contrast MRI data of 18 Chiari-I malformation patients and 21 controls. https://doi.org/10.5281/zenodo.5338940. ^40^

This project contains the following extended data:

- Video file: 57.mp4

Data are available under the terms of the Creative Commons Attribution 4.0 International license (CC-BY 4.0).

## Software availability

Archived source code at time of publication: https://doi.org/10.5281/zenodo.5200009. ^20^

- File: analyze.py

License: MIT

Supplementary information: https://doi.org/10.5281/zenodo.5229173. ^19^

- File: README.txt

License: Creative Commons Attribution 4.0 International license (CC-BY 4.0).

## Consent

Written informed consent for publication of the patients details and their images was obtained from the patients.
