# Hypothesis on the pathophysiology of syringomyelia based on analysis of phase-contrast magnetic resonance imaging of Chiari-I malformation patients

**DOI:** 10.12688/f1000research.72823.2

**Published:** 2023-08-03

**Authors:** Han Soo Chang

**Affiliations:** 1 Department of Neurosurgery, Tokai University, 143 Shimokasuya, Isehara, Kanagawa, 259-1143, Japan

**Keywords:** syringomyelia, Chiari malformation, pathophysiology, hypothesis, magnetic resonance imaging, phase-contrast

## Abstract

**Background:** Despite several hypotheses, our understanding of syringomyelia’s pathophysiology remains limited. The hypothesis proposed by Oldfield et al. suggests that piston-like movement of the cerebellar tonsils propels the cerebrospinal fluid (CSF) into the syrinx via the spinal perivascular space. However, a significant question remains unanswered: how does the CSF enter and stay in the syrinx, which has a higher pressure than the subarachnoid space. In the current study, we attempted to verify Oldfield’s hypothesis using phase-contrast magnetic resonance imaging (MRI) data from patients with syringomyelia.

**Methods:** We analyzed phase-contrast MRI scans of 18 patients with Chiari-I malformation associated with syringomyelia, all of whom underwent foramen magnum decompression, and 21 healthy volunteers. We obtained velocity waveforms for CSF and brain tissue from regions of interest (ROI) set at the various locations. These waveforms were synchronized at the peak timing of downward CSF flow. We compared the preoperative patient data with the control data and also compared the preoperative patient data with the postoperative patient data.

**Results:** The syrinx shrank in 17 (94%) of the patients, and they experienced significant clinical improvement. When comparing pre- and postoperative MRI results, the only significant difference noted was the preoperative elevated velocity of the cerebellar tonsil, which disappeared post-surgery. The CSF velocities in the subarachnoid space were higher in the preoperative patients than in the controls, but they did not significantly differ in the postoperative MRI. The tonsillar velocity in the preoperative MRI was significantly lower than that of the CSF, suggesting that the elevated tonsillar velocity was more of an effect, rather than the cause, of the elevated CSF velocity.

**Conclusions: **Given these findings, a completely new paradigm seems necessary. We, therefore, propose a novel hypothesis: the generative force of syringomyelia may be the direction-selective resistance to CSF flow in the subarachnoid space.

## Introduction

Syringomyelia is a condition in which a fluid-filled cavity, known as syrinx, forms inside the spinal cord, leading to neurological symptoms. Numerous hypotheses have been proposed by various authors to explain the mechanism of syrinx formation. ^1^ ^–^ ^13^ However, these hypotheses frequently contradict each other, and none seems to fully explain the pathophysiology of syringomyelia.

The prevailing hypothesis, proposed by Oldfield *et al.*, ^14^ suggests that piston-like movements of the cerebellar tonsils create pressure waves in the cerebrospinal fluid (CSF), which then push the CSF into the syrinx via the perivascular space. Despite its appeal, this hypothesis remains to be verified and requires new experimental data for confirmation.

Above all, a fundamental question remains unexplained. It is evident, as confirmed by laws of physics ^15^ and direct measurements, ^7^ ^,^ ^16^ ^,^ ^17^ that the pressure inside the syrinx is higher than that of the CSF outside. However, no hypothesis has yet explained how CSF enters and remains in the syrinx, given that its pressure is higher than the outside subarachnoid space.

In this article, we present our analysis of phase-contrast MRI of patients with Chiari-I-related syringomyelia. The data defied interpretation based on conventional theories, suggesting the need for the development of a new theoretical framework.

## Methods

### Material

This study solely included patients with Chiari-I malformations who had associated syringomyelia in the cervical cord. The following patients were excluded: those with syringomyelia not related to Chiari-I malformation, those with syringomyelia with basal arachnoiditis, and those with Chiari-I malformation without syringomyelia.

This study, approved by the Institutional Review Board of Tokai University Hospital (No. 18-609), was a retrospective study on prospectively acquired data. Since January 2011, we have routinely incorporated phase-contrast studies into the cervical spine MRI of patients with Chiari-I malformation. The MRI studies were performed before surgery and at each postoperative follow-up visit, namely, at six months, one year, and then annually thereafter. These MRI studies used the same acquisition techniques described below.

From January 2011 to April 2019, a total of eighteen eligible patients underwent foramen magnum decompression at our institution. These patients were consecutively enrolled in our study during this period. However, due to missing preoperative data for one patient, we analyzed 17 preoperative MRIs and 18 postoperative MRIs. In addition, we recruited 21 healthy volunteers from our hospital to ensure the mean age was comparable to that of the patients. These volunteers underwent the same MRI studies.

### Surgery

All patients underwent foramen magnum decompression performed by the first author using the same surgical techniques, which consisted of a small suboccipital craniotomy of 2 × 2 cm, C1 laminectomy, and a Y-shaped dural incision followed by fascia patching. We did not perform intradural exploration, which was not necessary in the studied population with no basal arachnoiditis. Next, the freed suboccipital bone flap was repositioned over the decompressed dura and secured with titanium miniplates at an angle to avoid dural compression. Following this, the fascia patch was sutured on the edge of the bone flap using two tenting sutures. This manoeuvre maintained decompression by preventing invasion of postoperative scar tissue.

### MRI method

All magnetic resonance images were obtained using a 1.5 Tesla scanner (Achieva, Philips Medical System, Best, The Netherlands). At each MRI session, phase-contrast images were obtained in the midline sagittal plane, encoding cranio-caudal motion into intensity with a velocity encoding (VENC) of 10 cm/sec. Data acquisition was triggered by the QRS wave of the patient’s electrocardiogram, with the cardiac cycle divided into 25 or 35 segments. The detailed imaging parameters were as follows: TR 16 msec, TE 7.2 msec, flip angle 15 degrees, field of view 256 × 256, matrix 352 × 256, and slice thickness 5 mm.

**Figure 1.**
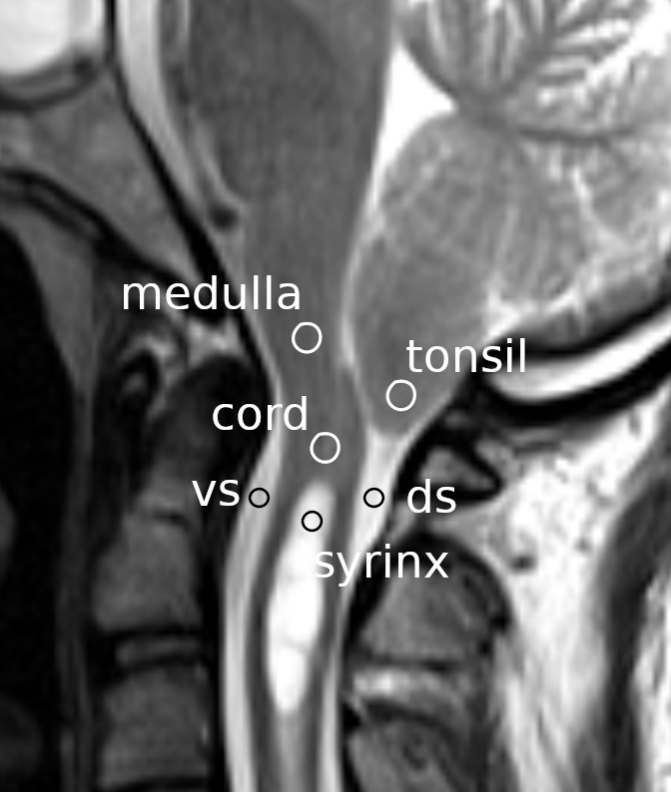
Regions of interest measured. Circles indicate the regions of interest on a mid-sagittal section of a magnetic resonance image at the craniovertebral junction. vs: ventral subarachnoid space; ds: dorsal subarachnoid space.

Each subject’s phase-contrast images were displayed on a computer monitor using an image-processing application ( ImageJ version 1.52a, National Institutes of Health, Bethesda, Maryland, United States (RRID:SCR_003070)). Circular-shaped regions of interest (ROIs) were set at the following locations ( Figure 1): the cerebellar tonsils, the spinal cord segment between the fourth ventricle and the syrinx, the ventral subarachnoid space at the level of the base of the odontoid process, the dorsal subarachnoid space immediately below the tonsil, the rostral portion of the syrinx cavity, and the medulla at the level slightly above the foramen magnum. For the control group, the same ROI settings were used except for the syrinx ROI, which was instead placed at the spinal cord segment at the level of the C3 vertebral body. The average flow speed of the pixels within each ROI was measured at each time point of the cardiac cycle. Consequently, six waveforms corresponding to the six ROIs were obtained for each MRI session and stored in a computer file.

#### Waveform synchronization

The obtained data began at the QRS wave of each patient’s electrocardiogram. However, the latency between the QRS wave and the initial rise of brain and CSF movements varied significantly among patients. This variability presented difficulties when we attempted to analyze the data in more detail. It was necessary to post-process the data so that the initial rise of the brain and CSF movements would be better synchronized. For this purpose, we employed the following synchronization technique.

Firstly, we defined the CSF trigger point as the time point when the ventral subarachnoid CSF started to move caudally i.e. where the CSF velocity most rapidly changed in the caudal direction. We could identify the CSF trigger point for each MRI session using the ventral subarachnoid CSF waveform from the data file. To account for varying numbers of time bins across MRI sessions, we increased the number of time bins per cardiac cycle to 50 using linear interpolation. We then shifted and synchronized the six waveforms using ring buffers, aligning the CSF trigger point to the midpoint of the waveform. The post-processed data is available in a data repository. ^18^ ^,^ ^19^ The software used for data analysis can be found in a software repository. ^19^ ^,^ ^20^

### Data analysis

We compared the following three groups: (1) Preoperative studies of Chiari-I patients, (2) Studies of normal volunteers, and (3) Postoperative studies of Chiari-I patients at the last clinical visit. For each group, we calculated the mean velocities at the six locations at each time point in the cardiac cycle, which was enabled by the aforementioned synchronization. The waveforms obtained were then plotted for each group.

We statistically compared the peak caudal velocity at each ROI. Comparisons were made between the control group and the preoperative Chiari-I patients, as well as between the preoperative and postoperative Chiari-I patients. For the ROI of the spinal cord, we also compared the peak rostral velocity in addition to the caudal velocity. This was because, as we describe below, a paradoxical rostral movement of the spinal cord was observed in Chiari-I patients.

For the statistical analyses, we used the unpaired t-test to compare the control group and preoperative Chiari-I patients, and the paired t-test to compare pre- and postoperative Chiari-I patients. For comparisons of tonsillar velocity and CSF velocities in the dorsal and ventral subarachnoid spaces, we used analysis of variance with Tukey’s post hoc multiple comparison test. P values smaller than 0.05 were considered statistically significant.

The following computer packages were used for the statistical analyses: R version 4.0.4 (R Project for Statistical Computing, RRID:SCR_001905) and RStudio version 1.2.1 (RStudio, RRID:SCR_000432).

## Results

### Clinical results

The mean age of the patients was 40.2 years with a standard deviation (SD) of 16.0. The patient group included fourteen females and four males. The mean age of the volunteers was 33.7 years with an SD of 9.8, and it consisted of seven females and fourteen males.

**Figure 2.**
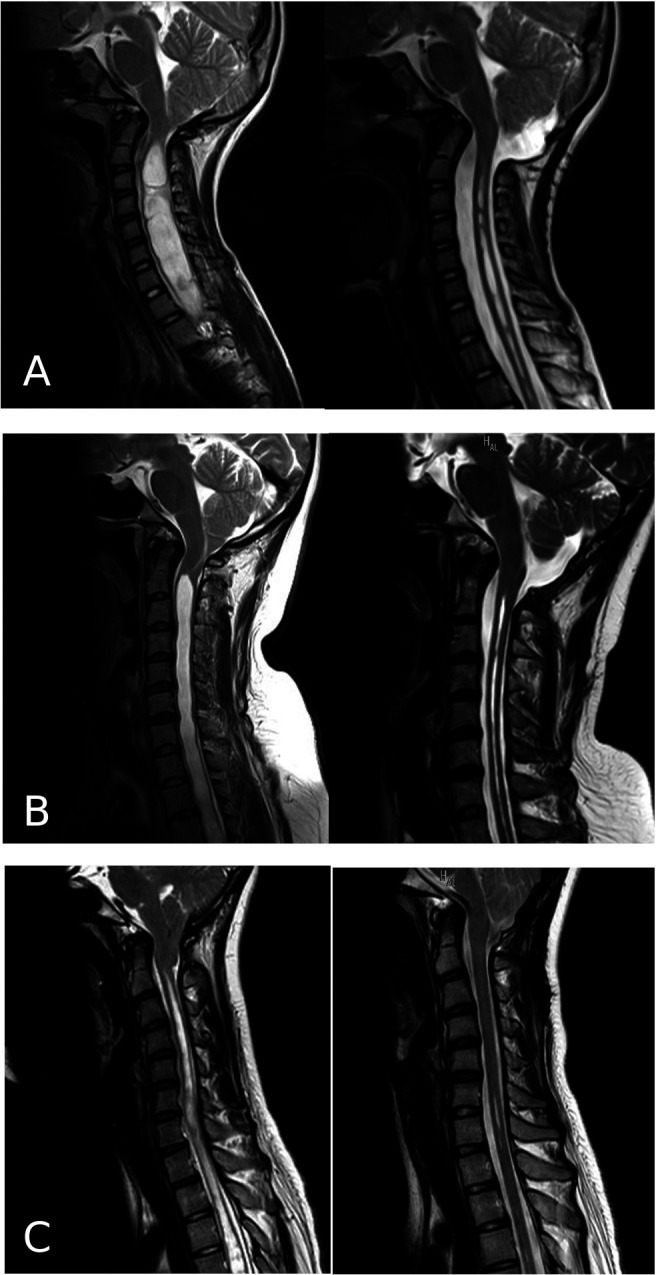
Pre- and postoperative cervical-spine MRI of three representative cases (A, B, and C). The left column shows the preoperative images, and the right column shows the postoperative images.

There were no complications related to the surgery. The average time from surgery to the final postoperative MRI was 633 days (SD 555, range 70–1700). The syrinx shrank in seventeen out of the eighteen patients (94%), which led to an improvement in preoperative symptoms ( Figure 2). In one patient, upper extremity pain persisted after surgery despite the syrinx shrinking.

### Velocity waveforms of preoperative Chiari-I patients

**Figure 3.**
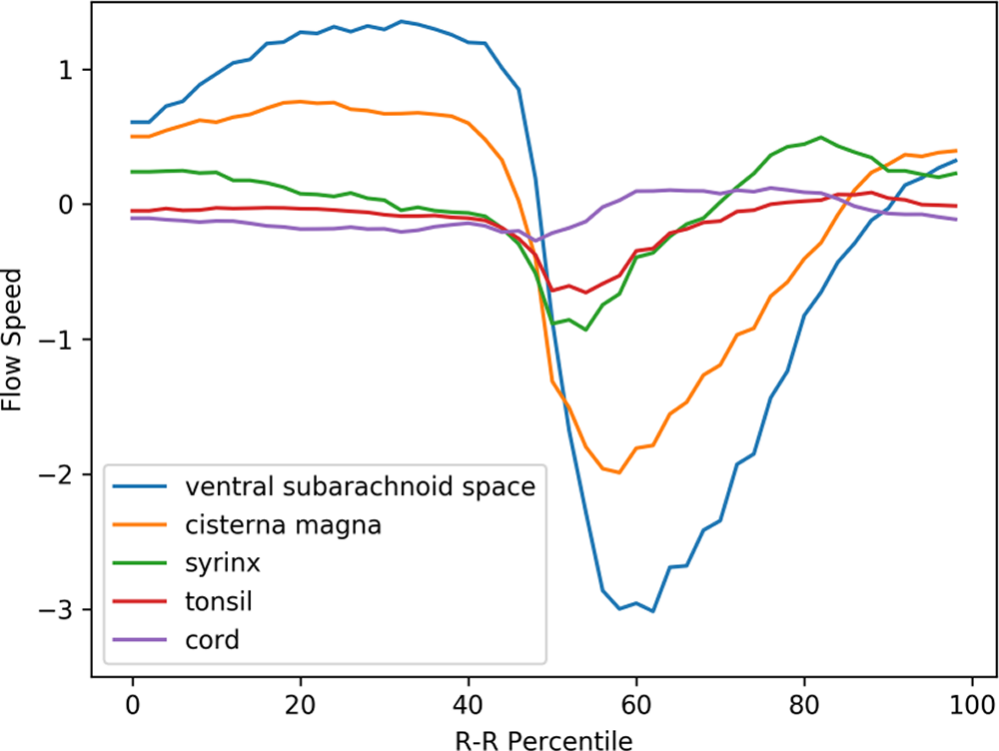
Mean velocity waveforms in preoperative Chiari-I patients. The abscissa represents one cardiac cycle, divided into 100 percentiles. The ordinate indicates the flow speed in cm/sec. The waveforms have been synchronized so that the point of maximal caudal acceleration of the ventral CSF is placed at the 50th percentile.

Figure 3 displays the mean velocity waveforms for five of the ROIs (excluding the medulla, which exhibited minimal movement) in preoperative Chiari-I patients. As demonstrated in the figure, the cerebellar tonsil (represented by the red line) moved rapidly in the caudal direction in sync with the rapid caudal flow of cerebrospinal fluid in the ventral and dorsal subarachnoid spaces (blue and yellow lines, respectively). The timing of this tonsillar movement was in synchrony with that of the CSF movement ( Figure 3).

The peak caudal velocity of the tonsils was significantly smaller than that of the CSF in the subarachnoid space. The mean peak caudal velocity (SD) was 0.76 (0.47) for the tonsils, 2.3 (1.7) for the dorsal subarachnoid space, and 3.5 (2.0) for the ventral subarachnoid. These differences were statistically significant (F = 13.9, p < 0.001). Tukey’s post hoc test revealed that each comparison pair was statistically significant, with p values of 0.02 for tonsil vs. dorsal subarachnoid space, <0.001 for tonsil vs. ventral subarachnoid space, and 0.05 for dorsal subarachnoid space vs. ventral subarachnoid space.

The syrinx fluid (represented by the green line in Figure 3) also exhibited a rapid caudal movement in synchrony with the CSF and the tonsil. This fluid movement occurred early in the timeline and was almost simultaneous with the caudal flow of the CSF (see Figure 3). There was no noticeable delay or phase shift between the start of the caudal syrinx fluid movement and that of the subarachnoid CSF movement ( Figure 3). Conversely, the reverse flow in the cranial direction began much earlier within the syrinx compared to the subarachnoid space (as shown in Figure 3).

**Figure 4.**
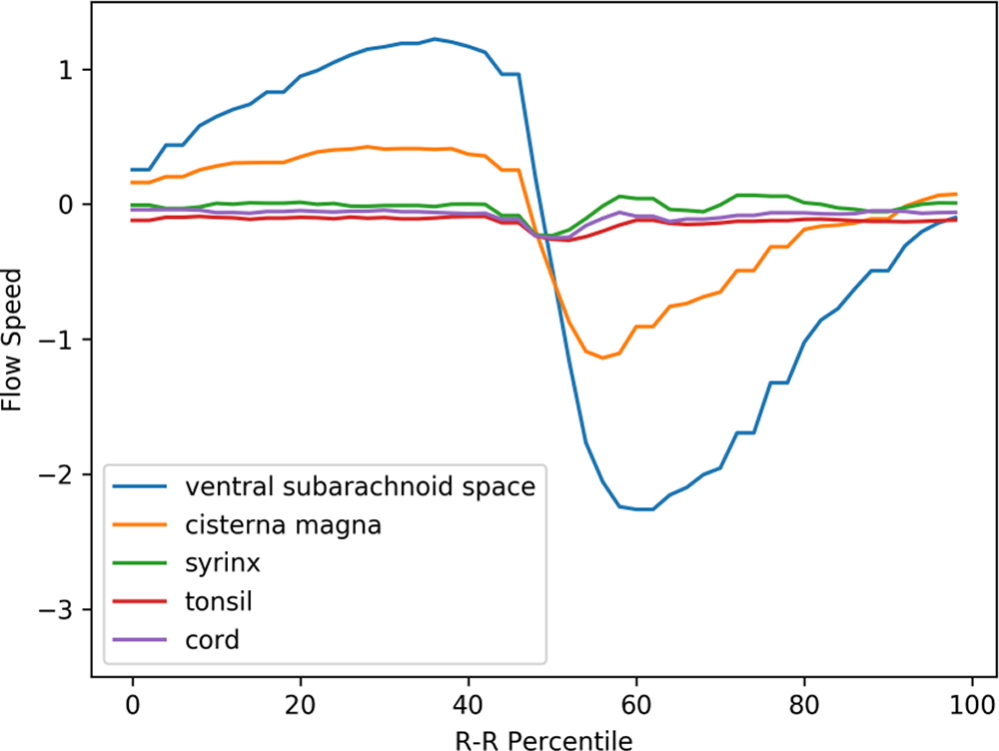
Mean velocity waveforms in controls. The same abscissa and ordinate as in Figure 3. (The legend *syrinx* denotes the spinal cord at the C5 level.)

A puzzling finding depicted in Figure 3 is the rostral movement of the upper cervical cord (represented by the purple line) occurring when all the other parts were moving caudally in patients. This paradoxical movement of the cord was observed in the controls (see Figure 4). This difference was statistically significant (refer to Table 1).

**Table 1.** Peak caudal velocity at the ROI in the three groups.

	Control	P (control vs. preop)	Preop	P (preop vs. postop)	Postop
tonsil	0.31 (0.14)	0.001*	0.76 (0.47)	0.002*	0.32 ( 0.39)
ventral SA	2.5 (0.77)	0.06	3.5 (2.0)	0.82	3.4 (1.3)
dorsal SA	1.3 (0.61)	0.04*	2.3 (1.7)	0.34	1.9 (1.1)
syrinx	0.34 (0.19)	0.001*	1.4 (1.1)	0.24	1.1 (1.0)
cord (rostral)	−0.0087 (0.0085)	0.002*	−0.31 (0.25)	0.49	−0.46 (0.34)
cord (caudal)	0.34 (0.13)	0.06	0.51 (0.32)	0.31	0.39 (0.23)
medulla	0.31 (0.11)	0.40	0.34 (0.25)	0.13	0.31 (0.20)

### Comparison between preoperative Chiari-I patients and controls

The prominent movement of the cerebellar tonsils observed in preoperative Chiari-I patients (represented by the red line in Figure 3) was absent in controls (refer to the red line in Figure 4). The paradoxical rostral movement of the upper cervical cord (represented by the purple line in Figure 3) seen in patients was also barely noticeable in the controls (see the purple line in Figure 4). These differences were statistically significant (refer to Table 1). The CSF velocities in the subarachnoid space were also significantly larger in preoperative Chiari-I patients than in controls (refer to Table 1).

### Comparison between pre- and postoperative Chiari-I patients

**Figure 5.**
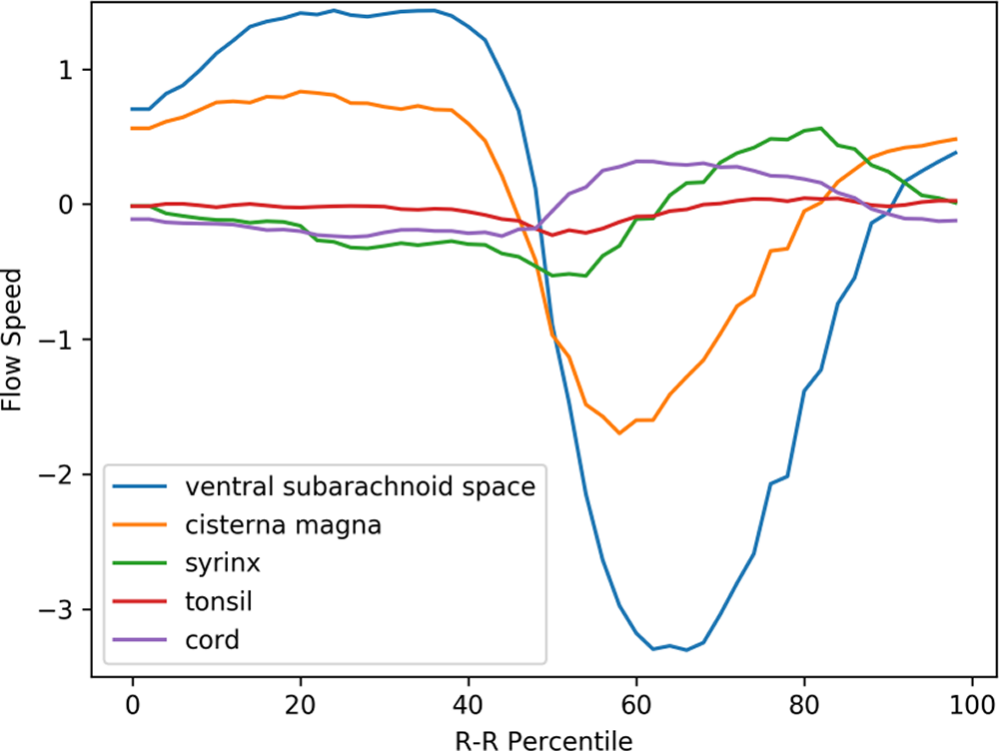
Mean velocity waveforms in postoperative Chiari-I patients. The same abscissa and ordinate as in Figures 3 and 4.

**Figure 6.**
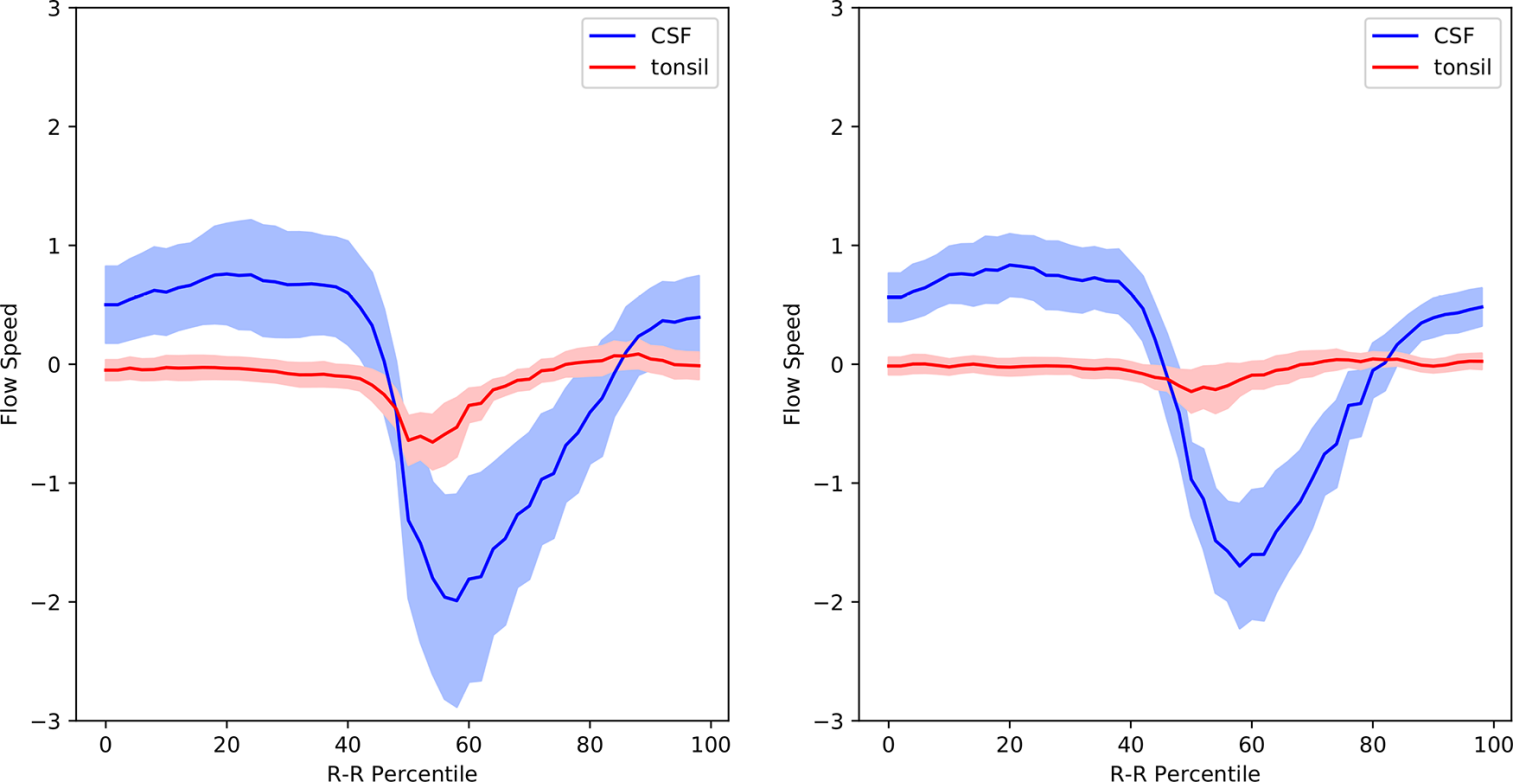
Velocity waveforms of the tonsil and the dorsal CSF in pre- and postoperative Chiari-I patients. The mean value is shown together with the 95% confidence intervals.

Figure 5 shows the postoperative mean velocity waveforms. The most notable postoperative change was the disappearance of tonsillar movement. Conversely, the velocity profiles of the other areas analyzed did not significantly deviate from the preoperative profiles (see Figures 3, 5, and Table 1). Figure 6 presents the velocity waveforms of the tonsil and the dorsal CSF extracted from Figures 3 and 5, along with their 95% confidence intervals. It is evident that the tonsillar velocity was significantly smaller than the CSF velocity, and despite the postoperative disappearance of tonsillar movement, the CSF velocity did not change significantly (refer to Figure 5 and Table 1).

## Discussion

### Summary of our findings

Our results can be summarized as follows.

1. The caudal velocity of the cerebellar tonsils was significantly elevated in preoperative Chiari-I patients, and it returned to normal postoperatively, in sync with syrinx shrinkage.
2. This preoperative tonsillar velocity was significantly lower than the velocity of the CSF.
3. There was no significant difference in the CSF velocity in the subarachnoid space between the pre- and postoperative studies.

The first point aligns well with previous studies. ^7^ ^,^ ^10^ ^,^ ^21^ ^–^ ^27^ The elimination of this elevated tonsillar velocity was the only parameter that changed post-surgery ( Figures 3, 5, and Table 1). Given that the syrinx shrank in 94% of the patients after surgery, this suggests a strong correlation between the elevated tonsillar velocity and syrinx generation.

The second point, which has not been reported in previous studies, carries theoretical importance. According to the hypothesis of Oldfield *et al*., ^7^ ^,^ ^14^ the piston-like movement of the tonsils generates pressure waves in the spinal subarachnoid CSF, which drives the CSF into the syrinx via the cord’s perivascular space. However, our data clearly demonstrated that the velocity of the tonsil was much smaller than that of the CSF ( Figure 6). It is challenging to envision how an object moving slower can be the source of a faster moving one. A completely different theoretical paradigm may be needed.

The third point is also important, despite the conflicting results found in the literature on this topic. ^7^ ^,^ ^26^ ^,^ ^27^ If we follow the postulation of Oldfield et al., which suggests that enhanced CSF pressure waves generate a syrinx, then postoperative syrinx shrinkage should be associated with a decreased CSF velocity in the subarachnoid space. This may also indicate the necessity for a novel theoretical paradigm.

A more intuitive interpretation of our results might be as follows: In Chiari-I patients, the decreased cross-sectional area of the subarachnoid space at the craniovertebral junction increases resistance to CSF flow. As a result, the velocity of the CSF at the craniovertebral junction was elevated because of the so-called Venturi effect. ^15^ Thus, the piston-like movement of the cerebellar tonsil might be more accurately seen as a result, not the cause, of this increased CSF velocity. Our surgical procedure expanded the cross-sectional area sufficiently to mitigate the elevated tonsillar movement. However, since its effect was only partial, the elevated CSF persisted. It should also be noted that the caudally displaced tonsils further reduce the cross-sectional area. Thus, the vertically moving tonsils create a mechanism resembling a ball valve: the resistance to the caudal flow becomes larger than that to the rostral flow.

### Towards a novel theoretical paradigm

As previously stated, we encountered difficulty interpreting our results within the confines of the current theoretical framework. Moreover, no existing theory adequately explains the paradoxical behavior of CSF entering and remaining in the syrinx despite the pressure gradient. Accordingly, we believe the situation warrants a novel theoretical approach. In subsequent sections, we first aim to examine the prerequisites that such a new theory must satisfy. Following this, we will propose our innovative hypothesis on the pathophysiology of syringomyelia. We offer a preemptive note of caution: this portion of the discussion is speculative and lacks direct support from our data. However, we felt it important to present one potential solution to the aforementioned theoretical challenges.

### Premises of the theory

We propose following three prerequisites for a theoretical understanding of syringomyelia.

1. The syrinx fluid originates from the CSF, and a channel exists that connects the syrinx cavity to the subarachnoid space.
2. The central canal cannot be dismissed as a potential candidate for this channel.
3. There must be some form of a one-way-valve mechanism in place to maintain the expanded state of the syrinx.

There is a significant amount of evidence supporting the first prerequisite. The composition of the syrinx fluid is the same as that of the CSF. ^28^ Intrathecally administered contrast or tracer materials readily enter the syrinx cavity. ^29^ ^–^ ^31^ Recently, Heiss *et al*., quantitatively analyzed the accumulation of intrathecally administered contrast material in non-tumor-related syrinxes. ^30^

The second prerequisite, however, is contentious and warrants detailed discussion in the next section.

### The central canal

Gardner and Angel ^1^ and Williams ^32^ initially hypothesized that CSF enters the syrinx through a patent central canal. However, this theory has recently fallen out of favor for several reasons.

1. MRI scans do not typically show a connection between the fourth ventricle and the syrinx in most cases.
2. Autopsy studies of syrinx patients have apparently shown minimal correlation between the syrinx and the central canal.
3. Autopsy studies have shown that the central canal gradually becomes obliterated as one ages.

The first point might not hold a valid argument. The diameter of the central canal is approximately 100 μm. ^33^ Given the current resolution of MRI scans, it is difficult to clearly display a channel of this size. ^34^ Therefore, the inability of MRI scans to illustrate the connection between the fourth ventricle and the syrinx does not necessarily prove or disprove the existence of such a channel.

Regarding the second point, Milhorat *et al*., ^35^ published an extensive autopsy series involving 105 syrinx patients. The authors classified the cases into 47 communicating and 23 noncommunicating syrinxes, based on the MRI findings. The remaining 35 cases were syrinxes of various etiologies. Of these 23 noncommunicating syrinxes, 70% extended rostrally to a stenotic central canal, while 30% extended to a patent central canal. As per the author’s descriptions, the stenotic central canals did not appear to be obliterated. To simplify, 100% of the noncommunicating syrinxes rostrally continued to a patent central canal. Therefore, these findings suggest, rather than refute, a potential role of the central canal in the pathogenesis of syringomyelia.

With regards to the third point, the central canal was traditionally believed to be occluded in human adults. ^34^ However, recent studies indicate that this occlusion is gradual process related to aging. ^33^ ^,^ ^36^ Newman *et al*., ^36^ found in their autopsy study of 60 cases that the central canal remained patent up until the fourth decade of life, while Yasui *et al*., ^33^ observed that occlusion of the central canal occurred at slightly earlier ages. In their study of 232 autopsy cases, Milhorat *et al*. reported that occlusion of the central canal was evident in only four individuals. ^37^ Storer *et al*., ^34^ suggested that”studying the morphology of the central canal using only histological sections” is challenging due to its three-dimensional nature, and proposed a computerized 3-D method for assessment. Considering that Chiari-I malformation typically occurs in the pediatric population and relatively early adulthood, ^3^ ^,^ ^38^ the potential role of a patent central canal in the pathogenesis of syringomyelia cannot be dismissed.

### One-way valve mechanism

The third assumption posits that a one-way valve mechanism is necessary to explain the generation and maintenance of syringomyelia. This idea has been proposed by previous authors. ^6^ ^,^ ^39^ However, the concept of the one-way valve mechanism has not been properly integrated into earlier theories of syringomyelia. Nevertheless, this concept might prove to be crucial if we consider the following points. Firstly, basic physical laws indicate that the syrinx, in its distended state, has a higher internal pressure than external. ^15^ It is intuitively understood that the internal pressure must counteract the elastic force of the syrinx wall, in addition to the external pressure. A few experiments with direct measurement of the syrinx pressure have proven that interior pressure was higher than the outside pressure. ^7^ ^,^ ^17^ ^,^ ^39^

Therefore, any theory of syringomyelia assuming a CSF channel between the syrinx and the subarachnoid space must account for the mechanism by which the syrinx maintains its expanded state against the pressure gradient. Without this mechanism, CSF would flow from the higher-pressure syrinx cavity to the lower-pressure subarachnoid space until the syrinx collapses and the pressure gradient is equilibrated. The assumption of a one-way valve existence appears to be a plausible solution to this problem.

### Hypothesis

Our hypothesis on the pathophysiology of syringomyelia can be summarized as follows:

1. There is a communication channel between the fourth ventricle and the syrinx, most likely a patent central canal.
2. When a direction-selective resistance is present in the subarachnoid space, repetitive pressure waves across this resistance pump CSF through this channel, thereby creating a syrinx distally.
3. Decompression of the foramen magnum neutralizes this direction-selective resistance by eliminating the local compression, leading to the collapse of the syrinx.

The concept of the central canal functioning as a one-way valve was proposed by du Boulay *et al*., in 1974. ^6^ This long-neglected idea warrants reconsideration in light of the premises outlined above. The herniated tonsils in Chiari patients move in a piston-like manner, aligning with the cyclical CSF movements. We might equate this piston-like movement to the movement of a ball in a ball valve. In other words, the CSF encounters more resistance in the caudal direction than in the rostral direction. Data show that the velocity of the tonsils and the CSF near the craniovertebral junction has higher peak velocity in the caudal direction than in the rostral direction ( Figure 3). As per the Venturi effect, higher velocity signifies higher resistance, suggesting there is a unidirectional resistance to the CSF flow at the craniovertebral junction; specifically, the resistance to the caudal CSF flow is greater than that to the rostral CSF flow. Williams *et al*. demonstrated this phenomenon and postulated that this unidirectional CSF resistance creates a *sucking* mechanism that generates syrinxes. ^2^ ^,^ ^32^ Our hypothesis bears resemblance to Williams’ idea, though a more detailed explanation is necessary. As demonstrated in our previous article, ^12^ when resistance to CSF flow in the subarachnoid space increases at a certain point, the transmural pressure in the central canal decreases in the downstream segment. Therefore, with reciprocating flows across a point with unidirectionally increased resistance, decreased intramural pressure will be repetitively generated in the central canal situated downstream to the increased resistance. This effectively creates a one-way valve mechanism in the central canal. We have supporting evidence for this concept from our simulation model ^11^ ^,^ ^12^ and are preparing to publish it.

Our hypothesis also clearly explains why limited decompression at the foramen magnum is effective in reducing the size of the syrinx. Localized decompression at the craniovertebral junction relieves cord compression and halts the piston-like movement of the tonsils along with the unidirectional CSF resistance. This disables the one-way valve function of the central canal. The fluid within the syrinx will then flow out in accordance with the pressure gradient, ultimately causing the syrinx to collapse.

This hypothesis resolves the problem concerning the interpretation of our phase-contrast data. The only difference in the postoperative phase-contrast data was the cessation of the tonsillar movement; there was no significant difference in the movement of the CSF in the subarachnoid space ( Figure 6). Therefore, an explanation was required to understand how the cessation of tonsillar movement caused the syrinx to shrink without significant changes in subarachnoid CSF movement. Our hypothesis explains this in a straightforward manner. The disappearance of the piston-like movement of the tonsils deactivates the one-way valve function of the central canal. The subarachnoid CSF movement plays no role in this causal relationship.

On the other hand, other hypotheses that consider the perivascular space as the CSF channel may encounter two major problems. First, these hypotheses might struggle to identify a one-way valve mechanism. There are no structures along the perivascular space that could function as a one-way valve. However, without presuming a one-way valve mechanism, the CSF in the syrinx would flow out due to the pressure gradient between the syrinx and the subarachnoid space. Second, even if there is a one-way valve in the perivascular space in the spinal cord, it is unclear how this valve would cease to function following simple decompression at the craniovertebral junction. Answering this question could pose a challenge. Therefore, in our opinion, the theories based on the perivascular space present a serious theoretical problem.

### Limitations and future prospects

The number of patients in this study was relatively small. Although statistically significant results were obtained, they should be interpreted with a certain degree of caution. While our hypothesis circumvented the difficulties of existing hypotheses and reasonably explained the data we gathered, it still lacks sufficient direct evidence to assert its truthfulness. Thus, our hypothesis thus remains as such until further supporting evidence is obtained in the future. However, it will serve as a working hypothesis for future syringomyelia studies. The exact mechanism of how the one-way valve appears in the central canal when the external CSF movement is blocked in one direction is not described in this report. A detailed explanation using computer simulation will soon be published. If a similar type of CSF movement blockage caused by the cerebellar tonsil occurs in other locations in the spinal canal, syrinxes could potentially be generated using the same mechanism. Therefore, it is possible that our hypothesis could be extended to explain the pathophysiology of syringomyelia associated with arachnopathy. ^40^

## Conclusions

The results of our phase-contrast data, obtained from Chiari-I patients, suggest a strong correlation between tonsillar movement and syrinx formation. However, this relationship does not appear to be mediated by CSF pressure waves in the subarachnoid space, which contradicts the existing hypotheses regarding the pathophysiology of syringomyelia. These findings imply the need for a new theoretical framework. We, therefore, propose a novel hypothesis for syrinx generation: the direction-selective resistance to CSF flow in the subarachnoid space creates a one-way-valve-like mechanism in the intraspinal channel. This could potentially resolve one the theoretical dilemma of how CSF enters and remains in the syrinx, despite it having a higher pressure than the surrounding subarachnoid space.

## Data availability

### Underlying data

Dryad: Underlying data ‘Phase-contrast MRI data of 18 Chiari-I malformation patients and 21 controls’. https://doi.org/10.5061/dryad.37pvmcvm0. ^18^

• Data files: encoded_data.json

Data are available under the terms of the Creative Commons Zero “No rights reserved” data waiver (CC0 1.0 Public domain dedication).

### Extended data

Zenodo: Extended data ‘Phase-contrast MRI data of 18 Chiari-I malformation patients and 21 controls. https://doi.org/10.5281/zenodo.5338940. ^41^

This project contains the following extended data:

• Video file: 57.mp4

Data are available under the terms of the Creative Commons Attribution 4.0 International license (CC-BY 4.0).

## Software availability

Archived source code at time of publication: https://doi.org/10.5281/zenodo.5200009. ^20^

• File: analyze.py

License: MIT

Supplementary information: https://doi.org/10.5281/zenodo.5229173. ^19^

• File: README.txt

License: Creative Commons Attribution 4.0 International license (CC-BY 4.0).

## Consent

Written informed consent for publication of the patients details and their images was obtained from the patients.
