# Supplementary material for: Hypothesis on the pathophysiology of syringomyelia based on analysis of phase-contrast magnetic resonance imaging of Chiari-I malformation patients
Source: F1000Res. 2023 Aug 3;10:996. Originally published 2021 Oct 1. [Version 2] doi: 10.12688/f1000research.72823.2 (PMC10450261; doi:10.12688/f1000research.72823.2)
Supplement: Peer review history

# Peer review: Reviewer response for version 2

**DOI:** 10.5256/f1000research.154031.r193500

**Published:** 2023-08-24

**Authors:** Yuichiro Ohnishi

**Affiliations:** 1 National Cerebral and Cardiovascular Center, Osaka, Japan

I appreciate this opportunity to review this paper. I say it is accepted for indexing.

Is the work clearly and accurately presented and does it cite the current literature?

Partly

If applicable, is the statistical analysis and its interpretation appropriate?

Partly

Are all the source data underlying the results available to ensure full reproducibility?

Partly

Is the study design appropriate and is the work technically sound?

Partly

Are the conclusions drawn adequately supported by the results?

Partly

Are sufficient details of methods and analysis provided to allow replication by others?

Partly

Reviewer Expertise:

Neurosurgery

I confirm that I have read this submission and believe that I have an appropriate level of expertise to confirm that it is of an acceptable scientific standard.

# Peer review: Reviewer response for version 2

**DOI:** 10.5256/f1000research.154031.r193499

**Published:** 2023-08-09

**Authors:** Petra M. Klinge

**Affiliations:** 1 Brown University, Providence, USA

I have no further comments to make. I can be accepted as it is.

Is the work clearly and accurately presented and does it cite the current literature?

Yes

If applicable, is the statistical analysis and its interpretation appropriate?

I cannot comment. A qualified statistician is required.

Are all the source data underlying the results available to ensure full reproducibility?

Yes

Is the study design appropriate and is the work technically sound?

Yes

Are the conclusions drawn adequately supported by the results?

Partly

Are sufficient details of methods and analysis provided to allow replication by others?

Yes

Reviewer Expertise:

Chiari, syringomyelia, tethered cord, hydrocephalus

I confirm that I have read this submission and believe that I have an appropriate level of expertise to confirm that it is of an acceptable scientific standard.

# Peer review: Reviewer response for version 1

**DOI:** 10.5256/f1000research.76427.r163449

**Published:** 2023-02-27

**Authors:** Yuichiro Ohnishi

**Affiliations:** 1 National Cerebral and Cardiovascular Center, Osaka, Japan

This manuscript investigated the differences of velocity waveforms of the CSF in various craniocervical locations between the preoperative and control cases, as well as between the preoperative and postoperative cases. Author described that the pressure inside the syrinx is higher than that of the CSF outside. There is the current prevailing hypothesis assumes that the piston-like movement of the cerebellar tonsils drives the CSF into the syrinx through the spinal perivascular space. Author also mentioned that a major unexplained problem is how CSF enters and remains in the syrinx that has a higher pressure than the subarachnoid space. Currently, it still remains unknown how CSF enters and remains in the syrinx, which has higher pressure than the outside subarachnoid space. Author propose a new hypothesis on the pathophysiology of syringomyelia.

 This study is the single institution and small sample size. Introduction is compact. I think author’s surgery is solid. Discussion is too speculatively written. Overall, I am impressed with the author's hypothesis. Results showed that the postoperative tonsillar velocity was significantly improved. While, there was no significant changes of subarachnoid CSF, spinal cord, and medulla in postoperative velocity. The abnormal tonsillar movement disappeared after surgery. However, results were inadequate to answer author's question. The pressure, CSF velocity, tonsillar movement, anatomical connection to syrinx, and other factors are implicated in Chiari-I malformation.

 My main concerns are below. 1) What is the reason that the velocity of postoperative CSF was not changed significantly. 2) The CSF velocity has the possibility to decrease the pressure of syrinx? 3) If so, you can explain this mechanism?

Is the work clearly and accurately presented and does it cite the current literature?

Partly

If applicable, is the statistical analysis and its interpretation appropriate?

Partly

Are all the source data underlying the results available to ensure full reproducibility?

Partly

Is the study design appropriate and is the work technically sound?

Partly

Are the conclusions drawn adequately supported by the results?

Partly

Are sufficient details of methods and analysis provided to allow replication by others?

Partly

Reviewer Expertise:

Neurosurgery

I confirm that I have read this submission and believe that I have an appropriate level of expertise to confirm that it is of an acceptable scientific standard, however I have significant reservations, as outlined above.

# Response

**Published:** 2023-08-04

**Authors:** Hans Soo Chang

**Affiliations:** Tokai University, Japan

Thank you very much for reviewing our manuscript.

 We have revised our manuscript, improving English expressions throughout. We have also rewritten the discussion to ensure it has a better logical structure.

 As we stated in the discussion, our data contradicts existing hypotheses. Moreover, the hypotheses that propose CSF enters the syrinx via the perivascular space face a significant theoretical challenge: they need to explain how CSF enters and remains in the syrinx against the pressure gradient (with syrinx pressure being higher than the surrounding subarachnoid space). We believe that a complete overhaul of the theoretical framework is needed. We must construct our theory from the ground up.

 In this context, we have offered our hypothesis as an attempt to overcome this theoretical difficulty. We clearly stated in the discussion that our hypothesis is speculative and is not directly supported by our data. However, given the current theoretical impasse, we believe that presenting our hypothesis is justified, as it could serve as a working hypothesis, stimulating future studies.

 The underlying mechanism of our theory is presented in our revised manuscript more clearly. For further clarification, we have conducted a computer simulation study of CSF dynamics in the spine to support our theory. We have drafted a manuscript and submitted it to a journal. It provides a more detailed explanation of our hypothesis. Unfortunately, it is still in the review phase, so we cannot present a citation at this time.

 We believe that our manuscript has improved significantly thanks to the reviewer's comments. We would be grateful if the reviewer could take the time to review our revised manuscript.

# Peer review: Reviewer response for version 1

**DOI:** 10.5256/f1000research.76427.r161190

**Published:** 2023-02-06

**Authors:** Petra M. Klinge

**Affiliations:** 1 Brown University, Providence, USA

**Editorial Note from F1000Research – 27 ^th^ February 2023: A previous version of this peer review report raised concerns about the methodology and data. Following further review of the data, the reviewer chose to withdraw these concerns. The approval status has been changed from Not Approved to Approved with Reservations**

 The author proposes a new concept of syringomyelia formation in CM based on a tailored MRI simulation. The weight is on the MRI methodology that has been published and presented previously and is available in the Data Availability section of the article. Given that, the data suggest an additional intriguing hypothesis of syrinx formation when comparing Chiari to controls and pre and post-surgery. The authors propose a one-way valve mechanism because that is the only way to explain syrinx formation and reduction based on their finding of reduced tonsil velocity and unchanged increased CSF velocity in the spinal SAS post-surgery. Naturally, there is no anatomical or other hard evidence available to show that the one-way valve mechanism is anatomically relevant and existing, but given the MRI observations and calculations in this patient series, even though speculative of nature and not the proof or implementation of a new concept, it is a reasonable addition to the pathophysiology of syringomyelia, which is still under scrutiny. I would only ask the authors to be mindful, when they say " that their hypothesis contradicts and or is a disproof of pre-existing theories"  because findings were interpreted based on different methods, tests and imaging algorithms. They should make a more balanced statement in this regard.

 The author must avoid a narrative style “ I have…” , “ I conducted…” , “ I did and decided...”. The entire paper is more a narrative style, particularly when detailing the methods.

 The MRI method may be reviewed by an MRI physicist or neuroradiologist. Also, the statistical methods need to be reviewed with a statistician as the paired and unpaired T-test with Turkey post-hoc seem too crude. ANOVA testing or multivariate testing seems more appropriate.

Is the work clearly and accurately presented and does it cite the current literature?

Yes

If applicable, is the statistical analysis and its interpretation appropriate?

I cannot comment. A qualified statistician is required.

Are all the source data underlying the results available to ensure full reproducibility?

Yes

Is the study design appropriate and is the work technically sound?

Yes

Are the conclusions drawn adequately supported by the results?

Partly

Are sufficient details of methods and analysis provided to allow replication by others?

Yes

Reviewer Expertise:

Chiari, syringomyelia, tethered cord, hydrocephalus

I confirm that I have read this submission and believe that I have an appropriate level of expertise to confirm that it is of an acceptable scientific standard, however I have significant reservations, as outlined above.

# Response

**Published:** 2023-08-04

**Authors:** Hans Soo Chang

**Affiliations:** Tokai University, Japan

Thank you very much for reviewing our manuscript.

 We have improved the English expressions throughout the entire manuscript as suggested by the reviewer.

 We have also enhanced the explanation of the synchronization method described in our manuscript. We believe that it has become much more understandable. Our procedure merely involves synchronizing the waveforms obtained from each individual. This is simply a post-processing of data acquired from the MRI and has no connection with MRI acquisition techniques. We believe there is no need for a physicist to review our manuscript.

 Regarding the statistics, it's important to note that we carried out two distinct comparisons: (1) preoperative Chiari patients versus controls, and (2) pre- and postoperative studies of Chiari patients. In the first comparison, the data consist of two separate series, while in the second comparison, the data represent repeated measures from the same individuals. We believe it's entirely reasonable to conduct t-tests individually for these two comparisons. However, if we were, as the reviewer suggested, to perform a statistical analysis combining these three groups: pre- and postoperative Chiari patients and controls, we would face a problem as the data incorporate both individual and repeated measures. To overcome this issue, we would need to resort to a more advanced statistical analysis such as mixed-effects models. Nevertheless, we believe this would be excessive for the current situation. Therefore, we maintain that our approach of employing two separate t-tests is justified.

 We believe that our manuscript has improved significantly thanks to the comments from the reviewer. We would be grateful if she could take the time to review our manuscript again.
